# Intensive care unit mortality and cost-effectiveness associated with intensivist staffing: a Japanese nationwide observational study

**DOI:** 10.1186/s40560-023-00708-w

**Published:** 2023-12-04

**Authors:** Saori Ikumi, Takuya Shiga, Takuya Ueda, Eichi Takaya, Yudai Iwasaki, Yu Kaiho, Kunio Tarasawa, Kiyohide Fushimi, Yukiko Ito, Kenji Fujimori, Masanori Yamauchi

**Affiliations:** 1 Department of Anesthesiology and Perioperative Medicine, Tohoku University Graduate School of Medicine, Sendai, Japan; 2 AI Lab, Tohoku University Hospital, Sendai, Japan; 3 Experience Design and Alliance Section, Tohoku University Hospital, Sendai, Japan; 4 Department of Biodesign, Center for Research, Education, and Innovation, Tohoku University Hospital, Sendai, Japan; 5 Department of Intensive Care Unit, Tohoku University Hospital, Sendai, Japan; 6 Department of Clinical Imaging, Tohoku University Graduate School of Medicine, Sendai, Japan; 7 Department of Health Administration and Policy, Tohoku University Graduate School of Medicine, Sendai, Japan; 8 Department of Health Policy and Informatics, Tokyo Medical and Dental University Graduate School of Medicine, Tokyo, Japan; 9 College of Policy Studies, Tsuda University, Tokyo, Japan

**Keywords:** Intensive care unit, Mortality, Incremental cost-effectiveness ratio, Cost-effectiveness, High-intensity staffing

## Abstract

**Background:**

Japan has four types of intensive care units (ICUs) that are divided into two categories according to the management fee charged per day: ICU management fees 1 and 2 (ICU1/2) (equivalent to high-intensity staffing) and 3 and 4 (ICU3/4) (equivalent to low-intensity staffing). Although ICU1/2 charges a higher rate than ICU3/4, no cost-effectiveness analysis has been performed for ICU1/2. This study evaluated the clinical outcomes and cost-effectiveness of ICU1/2 compared with those of ICU3/4.

**Methods:**

This retrospective observational study used a nationwide Japanese administrative database to identify patients admitted to ICUs between April 2020 and March 2021 and divided them into the ICU1/2 and ICU3/4 groups. The ICU mortality rates and in-hospital mortality rates were determined, and the incremental cost-effectiveness ratio (ICER) (Japanese Yen (JPY)/QALY), defined as the difference between quality-adjusted life year (QALY) and medical costs, was compared between ICU1/2 and ICU3/4. Data analysis was performed using the Chi-squared test; an ICER of < 5 million JPY/QALY was considered cost-effective.

**Results:**

The ICU1/2 group (*n* = 71,412; 60.7%) had lower ICU mortality rates (ICU 1/2: 2.6% vs. ICU 3/4: 4.3%, *p* < 0.001) and lower in-hospital mortality rates (ICU 1/2: 6.1% vs. ICU 3/4: 8.9%, *p* < 0.001) than the ICU3/4 group (*n* = 46,330; 39.3%). The average cost per patient of ICU1/2 and ICU3/4 was 2,249,270 ± 1,955,953 JPY and 1,682,546 ± 1,588,928 JPY, respectively, with a difference of 566,724. The ICER was 718,659 JPY/QALY, which was below the cost-effectiveness threshold.

**Conclusions:**

ICU1/2 is associated with lower ICU patient mortality than ICU3/4. Treatments under ICU1/2 are more cost-effective than those under ICU3/4, with an ICER of < 5 million JPY/QALY.

**Supplementary Information:**

The online version contains supplementary material available at 10.1186/s40560-023-00708-w.

## Background

The observed effects of intensive care unit (ICU) staffing on patient outcomes are contradictory. Wilcox et al. suggested that high-intensity staffing was associated with lower ICU and in-hospital mortality, whereas 24-h in-hospital intensivist placement compared with daytime-only placement did not reduce ICU or in-hospital mortality [1]. In contrast, Costa et al. suggested that high-intensity daytime staffing and closed ICUs did not reduce in-hospital mortality [2]. The European and North American guidelines recommend high-intensity staffing and closed ICUs; however, further research is required to determine the optimal provision of scarce medical resources due to the current constraints on the availability and cost of intensivists on a 24-h basis [3].

The universal health insurance system in Japan covers all citizens through the public healthcare insurance scheme [4]. The public healthcare insurance scheme classifies ICUs into four types that can be divided into two categories according to the management fee charged per day: (1) ICU management fees 1 and 2 (ICU1/2) (equivalent to high-intensity staffing), which require at least two full-time ICU specialists, certified nurses, and clinical engineers, and (2) ICU management fees 3 and 4 (ICU3/4) (equivalent to low-intensity staffing), which require only a full-time physician (not necessarily an ICU specialist), with no requirement of certified nurses or clinical engineers (Additional file 1). The accreditation for calculating the management fees is conducted on a hospital basis, and hospitals must satisfy the medical staffing and facility criteria to receive the certification. Whether the patient receives treatments in ICU1/2/3 or 4 is determined by the facility at which the patient is admitted, regardless of the disease or the severity of the illness [4]. ICU1/2, which is introduced in 2014, charges at a higher rate than ICU3/4. Although cost-effectiveness analyses of an emergency department-based ICU have been previously reported [5], no cost-effectiveness analysis on ICU1/2 (high-intensity staffing) has been conducted.

The Diagnosis Procedure Combination (DPC) database is a large Japanese database of inpatient records collected for designated acute-care hospitals [6, 7]. The Sequential Organ Failure Assessment (SOFA) score is a standard severity score used to assess organ damage, with a total score of 0–24 to indicate severity. The SOFA score is based on physiological parameters (respiration, coagulation, circulation, kidney, liver, and central nervous system) and is an accepted clinical indicator when assessing patient care [8]. As a requirement for calculating management fees, the Japanese public insurance payment scheme has mandated that SOFA scores be reported in the DPC Database with ICU1/2 claims from April 2018 onwards and with ICU3/4 claims from April 2020 onwards. Fujimori et al. suggested that the SOFA score may support a more accurate physiological severity-based analysis of treatment effects, which had not been possible in the past [9]. However, there have been no previous reports of physiological severity-based analysis comparisons performed using the SOFA score for patients treated under the ICU management fees. Therefore, this study aimed to compare the clinical outcomes between ICU1/2 and ICU3/4 and evaluate the cost-effectiveness of ICU1/2 compared with that of ICU3/4, using 1 year of DPC data.

## Methods

### Ethics approval

This study was exempt from requiring ethical approval from the Institutional Review Board of the University of Tohoku (reference no. 2022-1-444). The requirement for informed patient consent was also waived because of the anonymized nature of the data. This study was reported in accordance with the Consolidate Health Economic Evaluation Reporting Standards 2022 (CHEERS 2022) checklists [10].

### Study design and data source

This retrospective observational study analyzed inpatient data from the DPC database in Japan. The DPC database contains the clinical and medical expenditure information of over seven million patients admitted annually to the hospital, collected from nearly 1100 healthcare facilities. The database includes the following data for all inpatients: age; sex; diagnostic record with the International Classification of Diagnosis, 10th Revision (ICD-10) code, admission type (emergency or elective), daily procedures recorded using Japanese medical procedure codes, SOFA score during ICU admission, and discharge status.

### Patient selection

ICU patients enrolled in the DPC database between April 2020 and March 2021 were included in this study. Patients with ICU management fees 1, 2, 3, or 4 were eligible. The exclusion criteria were as follows: (1) requirement of multiple intensive care treatments under ICU1, 2, 3, and/or 4 in the same admission period; (2) readmission to the ICU after having previously been discharged; (3) age < 15 years; (4) ICU admission > 14 days; (5) missing or unclear SOFA score data; and (6) the surgery date did not correspond with the recorded anesthesia date. The selected patients were categorized into two groups: patients who received ICU management fees 1 or 2 (ICU1/2 group) and those who received ICU management fees 3 or 4 (ICU3/4 group). The following baseline patient information was collected: age at admission, sex, information on surgery, admission type, academic hospital or non-academic hospital, and usage of blood transfusion therapy.

### Outcomes

#### Clinical outcomes

The following clinical outcomes were compared between the ICU1/2 and 3/4 groups: ICU all-cause mortality, in-hospital all-cause mortality, length of ICU stay, and length of hospital stay. Patients who were discharged alive and those who died in the ICU or hospital were included in the evaluation of the length of hospital stay.

#### Cost

The medical costs for each patient from the day of ICU admission to the day of ICU discharge were obtained from the DPC data. The medical costs covered procedures, surgeries, anesthesia, blood transfusion, drugs, and hospital fees but excluded service fees of meals, transportation, and family care. The costs (which does not include the cost of surgery and anesthesia) and the total costs (which include the cost of surgery and anesthesia) were determined. The difference in the total costs between the ICU1/2 and ICU3/4 groups was used to analyze cost-effectiveness. Cost-effectiveness was determined based on the total costs, because the intention was to be close to real-world settings. All costs were obtained in Japanese Yen (JPY).

#### Evaluation of cost-effectiveness

For patients who were alive at hospital discharge, the expected life expectancy after hospital discharge was calculated using the life expectancy table obtained from the Japanese Ministry of Health, Labour and Welfare [11]. Patients who died during their hospital stay were recorded as having zero life expectancy. The life expectancy of the ICU-discharged population was estimated to be shorter than that of the general population of the same age [12–14]. Based on previous reports, the reduction rates according to the age group (≤ 51, 52–63, 64–74, ≥ 75 years) for patients who were discharged from the ICU were set to 0.66, 0.67, 0.56, and 0.71, respectively [15, 16]. The value of 0.65 was used as the overall reduction rate for patients who were discharged from the ICU. This value was calculated using a weighted average of the respective reduction rates in the percentage of the population by age in the present study. These reduction rates were used to calculate the life-year gained (LYG) by multiplying the life expectancy by reduction rates, as shown in the following formula [17]:

$${\text{LYG}} = {\text{life expectancy after ICU discharge}} \times {\text{reduction rates}}.$$

Although a decline in the quality of life (QOL) of the patients after discharge from the ICU was expected, QOL cannot be assessed directly with the DPC data [18–25]. Therefore, LYG was multiplied with utility values (derived from the EuroQol 5-dimensions [EQ-5D]) to estimate quality-adjusted life-year (QALY), as shown in the following formula [26]:

$${\text{QALY}} = {\text{LYG}} \times {\text{utility values}}{.}$$

A systematic review and previous literature reported that the utility values of patients admitted to the ICU ranged from 0.63 to 0.81 (Additional file 2) [18–25]. In this study, the utility values for ICU stay, emergency surgery, elective surgery, and non-surgical settings were 0.63, 0.70, 0.71, and 0.61, respectively [25]. Although several studies reported utility values classified by ICU stay, emergency surgery, elective surgery, and non-surgery, the lowest utility values reported were used in this study [19, 25].

The incremental cost-effectiveness ratio (ICER) was calculated as the difference in the total costs of ICU stay divided by QALY, as shown in the following formula:

$${\text{ICER = }}\left( {{\text{the average cost of ICU stay of ICU1/2 group}} - {\text{the average cost of ICU stay of ICU3/4 group}}} \right){/}\left( {{\text{the average QALY of ICU1/2 group}} - {\text{the average QALY of ICU3/4 group}}} \right).$$

The cost-effectiveness cutoff was specified as an ICER value of < 5 million JPY/QALY, according to previous studies conducted in Japan [27, 28]. The cost-effectiveness of ICU1/2 compared with that of ICU3/4 was analyzed from a health policy perspective.

#### Discount

Costs were not discounted as only the intensive care expenses were considered. Similarly, the time point was not considered, as only intensive care can be objective.

#### Subgroup analysis

Subgroup analyses of clinical outcomes and costs were conducted. Patients were stratified into subgroups by (1) age (≦ 51, 52–63, 64–74, and ≥ 75 years); (2) type of admission (elective admission, emergency admission); (3) type of surgery (emergency surgery, elective surgery, and non-surgery); and (4) SOFA scores (0–2, 3–5, 6–8, 9–11, 12–14, 15–24). The ICU mortality rates, in-hospital mortality rates, and cost-effectiveness for ICU1/2 and ICU3/4 were then compared according to these subgroups. The SOFA scores were calculated to determine the impact of the severity of organ damage [9, 29]. A surgery was selectively defined as a surgical operation performed on the same day with anesthesia (including general, intravenous, epidural, and spinal anesthesia). Emergency surgery was defined as emergency admission and surgery. Elective surgery was defined as elective admission and surgery. The category of non-surgical operation was used when a patient was not registered for surgery. The SOFA scores on the first day of ICU admission were used.

### Sensitivity analysis

Several sensitivity analyses were conducted to investigate the validity of the main analysis. The adjustments mentioned above that were used for the calculation of LYG and QALY (0.65 and 0.63) were obtained from the published literature; however, they were based on old studies; hence, the values were likely to be inaccurate. The sensitivity analysis was performed to investigate this uncertainty for the calculated factors of LYG and QALY. In this scenario, the variation of ICERs was examined when the adjustment factors were changed. In the worst case, a sensitivity analysis was performed when reduction rates were 0.5 for the LYG calculation and 0.5 for the QALY calculation. In the best case, a sensitivity analysis was also performed when reduction rates were 0.8 for the LYG calculation and 0.9 for the QALY calculation. The sensitivity analysis set values to a range covering previously reported utility values for ICU stay of 0.63 to 0.81 (Additional file 2) [18–25]. The results were compared with the values obtained under the standard condition (basic case).

### Statistical analysis

All analyses were performed using Python (version 3.7.13) software. Continuous variables are expressed as means and standard deviations, while categorical variables are expressed as numbers and percentages. The Chi-squared test and Wilcoxon rank-sum test were used to compare the two groups. For all analyses, statistical significance was defined as *p* < 0.05.

## Results

### Patients

**Fig. 1:**
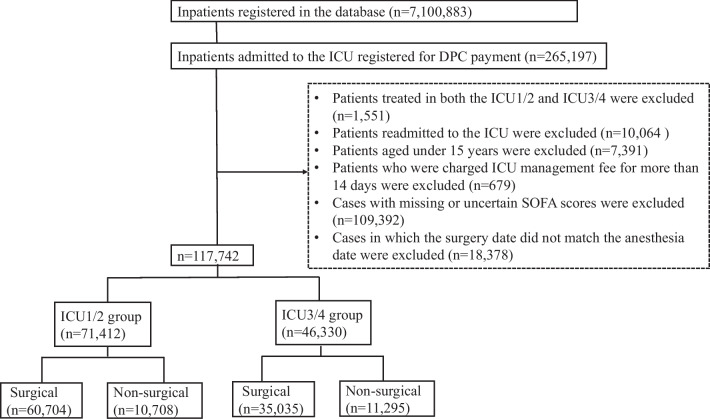
Flowchart of patient selection. *ICU* intensive care unit, *DPC* diagnostic procedure combination, *SOFA* Sequential Organ Failure Assessment

In total, 7,100,883 patients were enrolled in the DPC database between April 2020 and March 2021. Among these, 265,197 patients were admitted to the ICU under ICU management fees 1, 2, 3, and/or 4 during the study period. Thus, a total of 117,742 patients from 495 different hospitals were included in the analysis, with 71,412 patients (60.7%) in the ICU1/2 group and 46,330 (39.3%) in the ICU3/4 group (Fig. 1). Compared with the ICU3/4 group, the ICU1/2 group was younger (68.1 years vs. 70.3 years, *p* < 0.001), underwent a higher proportion of surgery (85.0% vs. 75.6%, *p* < 0.001), were more likely to be admitted in academic hospitals (42.8% vs. 12.2%, *p* < 0.001), had a lower proportion of emergency admission (36.3% vs. 46.8%, *p* < 0.001), and had higher SOFA scores (4.4 vs. 3.9, *p* < 0.001) (Table 1).

**Table 1:** Baseline patient characteristics

	Overall		ICU1/2 group		ICU3/4 group		*p* value
	(*n* = 117,742)	%	(*n* = 71,412, 60.7%)	%	(*n *= 46,330, 39.3%)	%	
Hospital	495		186	37.6	309	62.4	
Age (years)	69.0 (14.6)		68.1 (14.8)		70.3 (14.2)		< 0.001
≦51	14,990	12.7	9862	13.8	5128	11.1	< 0.001
52–63	17,921	15.2	11,321	15.9	6600	14.2	< 0.001
64–74	37,492	31.8	23,067	32.3	14,425	31.1	< 0.001
≧75	47,339	40.2	27,162	38.0	20,177	43.6	< 0.001
Male sex	70,369	59.8	42,951	60.1	27,418	59.2	< 0.001
Emergency hospital admission	47,602	40.4	25,917	36.3	21,685	46.8	< 0.001
Academic hospital	36,255	30.8	30,596	42.8	5659	12.2	< 0.001
Surgery	95,739	81.3	60,704	85.0	35,035	75.6	< 0.001
SOFA score	4.2 (3.7)		4.4 (3.7)		3.9 (3.8)		< 0.001
Blood transfusion therapy							
RBC transfusion	37,765	32.1	25,045	35.1	12,720	27.5	< 0.001
Plasma transfusion	23,451	19.9	15,982	22.4	7469	16.1	< 0.001
Platelet transfusions	14,706	12.5	10,283	14.4	4423	9.5	< 0.001

Data are presented as the number (frequency), or mean (SD)

*SD* standard deviation; ICU, intensive care unit; IQR, interquartile range; SOFA, Sequential Organ Failure Assessment

### Clinical outcomes

Table 2 presents the comparison of the clinical outcomes between the ICU1/2 and ICU3/4 groups. The ICU mortality rates were 2.6% and 4.3% in the ICU1/2 and ICU3/4 groups, respectively (*p* < 0.001). The in-hospital mortality rates were 6.1% and 8.9% (*p* < 0.001), and the length of ICU stay was 2.8 days and 2.7 days in the ICU1/2 and ICU3/4 groups (*p* < 0.001), respectively. The length of hospital stay was longer in the ICU1/2 group (27.0 days vs. 23.6 days, *p* < 0.001).

**Table 2:** Clinical outcomes and costs

	ICU1/2 group	ICU3/4 group	Difference	*p* value
	*n* = 71,412	*n* = 46,330		
Clinical outcomes				
ICU mortality rates	1870 (2.6%)	1983 (4.3%)		< 0.001
In-hospital mortality rates	4316 (6.1%)	4102 (8.9%)		< 0.001
Length of ICU stay, days	2.8 (3.1)	2.7 (3.0)		< 0.001
Length of hospital stay, days	27.0 (28.2)	23.6 (23.3)		< 0.001
Cost				
Cost (excluding surgery and anesthesia), JPY	571,106 (763,141)	397,756 (507,567)	173,350	< 0.001
Total cost, JPY	2,249,270 (1,955,953)	1,682,546 (1,588,928)	566,724	< 0.001
Life expectancy, years	19.29	17.35	1.93	
LYG, years	12.50	11.25	1.25	
QALY, years	7.87	7.08	0.79	
ICER, JPY/QALY			718,659	

Data represent number (frequency), or mean (SD). Costs are expressed in JPY

*SD* standard deviation, *ICU* intensive care unit, *LYG* life year gained, *QALY* quality-adjusted life-year, *ICER* incremental cost-effectiveness

### Cost-effectiveness ratio

Table 2 presents the calculation results of the average medical costs from the day of ICU admission to the day of ICU discharge. The costs per patient, excluding the costs of surgery and anesthesia, was 571,106 ± 763,141 JPY in the ICU1/2 group and 397,756 ± 507,756 JPY in the ICU3/4 group. The total costs per patient in the ICU1/2 group was 2,249,270 ± 1,955,953 JPY, whereas that in the ICU3/4 group was 1,682,546 ± 1,588,928 JPY. The difference of 566,724 JPY indicated that the ICU1/2 group had higher total medical costs than the ICU3/4 group. Table 2 also presents comparisons of life expectancy, LYG, and QALY. The life expectancy was 19.29 years and 17.35 years in the ICU1/2 and ICU3/4 groups, respectively, 1.93 years longer in the ICU1/2 group. LYG, which was calculated by multiplying life expectancy by 0.65, was 1.25 years longer in the ICU1/2 group than that in the ICU3/4 group. QALY, which was calculated by multiplying LYG by 0.63, was 0.79 years longer in the ICU1/2 group. The ICER obtained from the differences in cost and QALY was 718,659 JPY/QALY.

### Subgroup analysis

The results of the subgroup analysis of age, type of admission, type of surgery, and SOFA scores are shown in Table 3. The ICU mortality rates in ICU1/2 were lower than those in ICU3/4 for all age subgroups: ≤ 51 years (1.8% vs. 2.8%, *p* < 0.001), 52–63 years (1.9% vs. 3.1%, *p* < 0.001), 64–74 years (2.1% vs. 3.3%, *p* < 0.001), and ≥ 75 years (3.6% vs. 5.7%, *p* < 0.001). For all age subgroups, in-hospital mortality was also lower in ICU1/2 than in ICU3/4. Both the ICU mortality rates and in-hospital mortality rates in ICU1/2 were lower than those in ICU3/4 for emergency admission (ICU mortality rates: 6.3% vs. 8.4%,* p* < 0.001, in-hospital mortality rates: 14.1% vs. 17.1%, *p* < 0.001) subgroups but not for the elective admission subgroup. Meanwhile, the ICU mortality rates and in-hospital mortality rates in ICU1/2 were lower than those in ICU3/4 for the emergency surgery (ICU mortality rates; 3.1% vs. 3.9%, *p* < 0.001, in-hospital mortality rates; 9.6% vs. 11.2%, *p* < 0.001) and non-surgery (ICU mortality rates; 11.6% vs. 13.0%, *p* < 0.001, in-hospital mortality rates; 21.6% vs. 23.1%, *p* < 0.001) subgroups but not for the elective surgery subgroup. Further, for emergency surgery patients, the in-hospital mortality rates were lower for ICU1/2 than those for ICU3/4 in the subgroup of patients with SOFA scores of 0–2, 3–5, 6–8, and 9–11. For non-surgery patients, the in-hospital mortality rates were lower for ICU1/2 than those for ICU3/4 in the SOFA scores of 0–2, 3–5, 6–8, 9–11, and 12–14 subgroups. Among patients with SOFA scores of 0–2, in-hospital mortality rates was lower in the ICU1/2 than in the ICU3/4 in the emergency surgery and non-surgery subgroups, but there was no difference in the elective surgery subgroup. In the age subgroups, the lowest and highest ICERs were observed in the age ≤ 51 years subgroup (850,693 JPY/QALY) and in the age 64–74 years subgroup (4,584,021 JPY/QALY), respectively. In the admission type subgroups, ICER was higher in the elective admission subgroup (824,716 JPY/QALY) than in the emergency admission subgroup (782,439 JPY/QALY). In the surgery type subgroups, ICER was higher in the emergency surgery subgroup (776,991 JPY/QALY) than in the elective surgery subgroup (736,932 JPY/QALY). The ICER in the non-surgery subgroup was 432,369 JPY/QALY.

**Table 3:** Subgroup analysis of mortality and cost-effectiveness by information on age, admission, surgery, and SOFA scores

	ICU mortality				*p* value	In-hospital mortality				*p* value	Difference in cost (JPY)	Difference in life expectancy (years)	Difference in LYG (years)	Difference in QALY (years)	ICER (JPY/QALY)
	Number of deaths/number of patients, %					Number of deaths/number of patients, %									
	ICU1/2	%	ICU3/4	%		ICU1/2	%	ICU3/4	%						
Overall	1870/71,412	2.6	1983/46,330	4.3	< 0.001	4361/71,412	6.1	4102/46,330	8.9	< 0.001	566,724	1.93	1.25	0.79	718,659
Age, years															
Subgroup 1 (≦ 51)	177/9862	1.8	144/5128	2.8	< 0.001	387/9,862	3.9	248/5128	4.8	< 0.001	566,300	1.60	1.06	0.67	850,693
Subgroup 2 (52–63)	218/11,321	1.9	204/6600	3.1	< 0.001	537/11,321	4.7	375/6600	5.7	< 0.001	488,301	0.27	0.18	0.11	4,344,097
Subgroup 3 (64–74)	494/23,067	2.1	480/14,425	3.3	< 0.001	1203/23,067	5.2	945/14,425	6.6	< 0.001	488,134	0.30	0.17	0.11	4,584,021
Subgroup 4 (≧ 75)	981/27,162	3.6	1155/20,177	5.7	< 0.001	2234/27,162	8.2	2534/20,177	12.6	< 0.001	669,908	0.71	0.50	0.31	2,145,136
Admission type															
Elective admission	243/45,495	0.5	158/24,645	0.6	0.073	718/45,495	1.6	394/24,645	1.6	0.836	382,141	1.14	0.74	0.46	824,716
Emergency admission	1627/25,917	6.3	1825/21,685	8.4	< 0.001	3643/25,917	14.1	3708/21,685	17.1	< 0.001	713,846	2.23	1.45	0.91	782,439
Emergency surgery	486/15,840	3.1	420/10,743	3.9	< 0.001	1525/15,840	9.6	1208/10,743	11.2	< 0.001	605,377	1.71	1.11	0.78	776,991
SOFA scores															
Subgroup 1 (0–2)	25/4385	0.6	27/4010	0.7	0.547	97/4385	2.2	136/4010	3.4	< 0.001	365,369	2.04	1.32	0.93	394,081
Subgroup 2 (3–5)	48/4396	1.1	66/2946	2.2	< 0.001	253/4396	5.8	264/2946	9.0	< 0.001	478,963	2.60	1.69	1.18	404,594
Subgroup 3 (6–8)	74/3445	2.1	86/1866	4.6	< 0.001	315/3445	9.1	272/1866	14.6	< 0.001	502,951	2.27	1.48	1.03	486,621
Subgroup 4 (9–11)	137/2292	6.0	97/1198	8.1	0.017	411/2292	17.9	261/1198	21.8	0.006	507,200	2.02	1.31	0.92	552,667
Subgroup 5 (12–14)	130/1030	12.6	89/538	16.5	0.033	305/1030	29.6	180/538	33.5	0.118	566,486	1.46	0.95	0.67	850,518
Subgroup 6 (15–24)	72/292	24.7	55/185	29.7	0.222	144/292	49.3	95/185	51.4	0.665	786,602	2.58	1.67	1.17	671,005
Elective surgery	143/44,864	0.3	97/24,292	0.4	0.085	520/44,864	1.2	288/24,292	1.2	0.757	381,083	1.12	0.73	0.52	736,932
SOFA scores															
Subgroup 1 (0–2)	4/20,949	0.0	4/14,257	0.0	0.584	40/20,949	0.2	39/14,257	0.3	0.108	174,093	1.57	1.02	0.72	240,206
Subgroup 2 (3–5)	12/12,702	0.1	9/5527	0.2	0.211	78/12,702	0.6	56/5527	1.0	0.004	303,501	1.27	0.83	0.59	516,341
Subgroup 3 (6–8)	28/6366	0.4	10/2323	0.4	0.953	97/6,366	1.5	47/2323	2.0	0.106	365,490	1.78	1.15	0.82	445,860
Subgroup 4 (9–11)	29/3409	0.9	25/1537	1.6	0.015	121/3,409	3.5	64/1537	4.2	0.292	309,338	1.53	1.00	0.71	437,516
Subgroup 5 (12–14)	35/1174	3.0	30/530	5.7	0.008	108/1,174	9.2	56/530	10.6	0.376	348,303	0.79	0.51	0.36	961,209
Subgroup 6 (15–24)	35/264	13.3	19/118	16.1	0.008	76/264	28.8	26/118	22.1	0.168	1,451,111	1.13	0.73	0.52	2,782,327
Non-surgery	1241/10,708	11.6	1466/11,295	13.0	0.002	2,316/10,708	21.6	2606/11,295	23.1	0.010	398,879	2.33	1.51	0.92	432,369
SOFA scores															
Subgroup 1 (0–2)	43/2653	1.6	62/3781	1.6	0.953	111/2653	4.2	191/3781	5.1	0.001	242,197	3.56	2.31	1.41	171,521
Subgroup 2 (3–5)	116/3222	3.6	187/3391	5.5	< 0.001	375/3222	11.6	489/3391	14.4	< 0.001	443,260	3.18	2.07	1.26	351,592
Subgroup 3 (6–8)	251/2368	10.6	275/2051	13.4	0.004	562/2368	23.7	602/2051	29.4	< 0.001	564,818	2.97	1.93	1.18	479,695
Subgroup 4 (9–11)	297/1392	21.3	363/1095	33.2	< 0.001	548/1392	39.4	575/1095	52.5	< 0.001	625,128	3.70	2.40	1.47	426,560
Subgroup 5 (12–14)	302/709	42.6	350/665	52.6	< 0.001	428/709	60.4	482/665	72.5	< 0.001	471,555	2.24	1.45	0.89	531,997
Subgroup 6 (15–24)	232/361	63.7	229/312	73.4	0.007	292/361	80.2	267/312	85.6	0.066	654,238	0.80	0.52	0.32	2,054,148

*ICU* intensive care unit, *ICER* incremental cost-effectiveness ratio, *JPY* Japanese Yen, *LYG* life year gained, *QALY* quality-adjusted life years, *QOL* quality of life, *SOFA* Sequential Organ Failure Assessment

### Sensitivity analysis

Sensitivity analyses were performed to examine the impact of changing the adjustment factors used to estimate LYG and QALY from the life expectancy years. The results of the sensitivity analysis are presented in Table 4**.** The ICER was 407,480 JPY/QALY for the combination of 0.8 reduction rate and 0.9 utility value. The ICER for the combination of 0.5 reduction rate and 0.5 utility value was 1,173,541 JPY/QALY.

**Table 4:** Sensitivity analysis

	Reduction rate for LYG estimation	Utility value for QALY estimation	Difference in cost (JPY)	Difference in life expectancy (years)	Difference in LYG (years)	Difference in QALY (years)	ICER (JPY/QALY)
Case 1 (Basic case)	0.65	0.63	566,724	1.93	1.25	0.79	718,659
Case 2 (Best case)	0.8	0.9	566,724	1.93	1.55	1.39	407,480
Case 3 (Worst case)	0.5	0.5	566,724	1.93	0.97	0.48	1,173,541

*LYG* life year gained, *QALY* quality-adjusted life-year, *ICER* incremental cost-effectiveness ratio, *JPY* Japanese Yen

## Discussion

A systematic review concluded that providing higher intensive care in the ICU significantly reduces the mortality of emergency patients [30–35]. Neuraz et al. reported that the number of intensivists in an ICU significantly reduced the mortality of patients [31]. Endo et al. also suggested that the intensivists’ skills were associated with the mortality of ICU patients in acute-care situations [32]. The results of our study are consistent with those of previous studies. The mortality rates of ICU patients in ICU1/2, where the number of engaged certified intensivists was higher than that in ICU3/4, were significantly lower than that of patients in ICU3/4. Needleman et al. and Penoyer et al. also reported that the number of nurses was also associated with lower in-hospital mortality [33, 34]. Although the staffing criteria (nurse-to-patient ratio) is the same for ICU1/2 and ICU3/4 in our DPC system, ICU1/2 requires certified nurses. Our findings indicate that the skill level, as well as the number, of nurses may impact the reduction of ICU mortality rates. Although this factor has not been thoroughly investigated in previous studies, our result indicates that the presence of clinical engineers may also contribute to lower mortality rates as evidenced by the lower ICU and in-hospital mortality rates in ICU1/2, which employs clinical engineers, than in ICU3/4, which does not.

Our finding of lower ICU and in-hospital mortality rate in ICU1/2 than in ICU3/4 in the emergency surgery subgroup aligns with that of a previous study [35]. In the current study, the emergency surgery patients with SOFA scores of 0–2, 3–5, 6–8, and 9–11 had lower in-hospital mortality when admitted to ICU1/2. There was no significant difference in ICU and in-hospital mortality among elective surgery patients with SOFA scores of 0–2. As most postsurgical patients with mild general conditions are admitted to the ICU only for intensive monitoring without active intervention, a low-intensity model or high-dependency care unit may be sufficient for elective postsurgical patients with SOFA scores of 0–2 [3]. There are no cost-effectiveness studies comparing the effect between high- and low-intensity staffing on the ICU management fees. Shiroiwa et al. reported that medical interventions were cost-effective, with ICER values of < 5 million JPY/QALY gained [27, 28]. Based on this criterion, our research finding suggests that treatments in ICU1/2 are more cost-effective than those in ICU3/4 (718,659 JPY/QALY). ICU1/2 is associated with a higher fee than ICU3/4 because of its medical staffing and facilities requirements. In this study, the ICER was < 5 million JPY/QALY in the comparison between ICU1/2 and ICU3/4, suggesting that, from a health policy perspective, the cost-effectiveness criteria are satisfied. However, the Japanese Society of Intensive Care Medicine Board reports that more than 80% of hospitals accredited for ICU management fees (ICU1/2 and ICU3/4) have a generous nurse-to-patient ratio of 1:1.5, even if the staffing criterion is 1:2 [36]. More medical staff may be assigned in ICU1/2 than in ICU3/4, which may result in higher labor costs. From a hospital perspective, it could possibly mean that the medical fee for ICU 1/2 is not adequately proportionate, and cost-effectiveness is established with the disadvantage of a large medical expenditure in the hospital.

ICU survivors have a shorter life expectancy after discharge than healthy individuals. To account for this, LYG and QALY were calculated using reduction rates and utility values, based on previous studies. A sensitivity analysis was performed to determine the effect of this uncertainty on the values. The ICERs calculated for cases with higher values (0.8 and 0.9) and lower values (0.5 and 0.5) were 407,480 JPY/QALY and 1,173,541 JPY/QALY, respectively. These ICERs are below the acceptable threshold of ICER even in the worst case. With regard to subgroup analyses according to age, type of admission, type of surgery, and SOFA scores, ICERs were confirmed to be below the acceptable threshold in all subgroups. In ICU1/2- and ICU3/4-certified facilities, all patients are charged the same ICU management fees per day. Differentiating management fees according to patient backgrounds, such as the type of surgery (emergency, elective, or non-surgery) and SOFA score, is theoretically rational with respect to health economics. Further, it encourages hospitals to redistribute healthcare resources based on necessity. Although the assessment of QOL in ICU patients varies from discharge to a longer time point of 6 months to 12 years after ICU treatment [19, 22–25, 37–39], the timing of ICER assessment was set to a shorter time point setting from ICU admission to hospital discharge in this study. This approach could also be used to evaluate claims data from other countries and applied to international comparisons of healthcare economics. The long-term clinical outcomes, including QOL, of patients discharged from ICUs must be further investigated to determine cost-effectiveness in the intensive care setting.

Our study had several limitations. First, although a subgroup analysis was conducted, the presence of unadjusted confounding factors could not be eliminated. No adjustment for facility or region was performed. Second, patients with missing or unknown SOFA scores were excluded from the study, and unregistered or undeclared SOFA scores were more prevalent in the ICU3/4 group. Patients with ICU stay > 14 days were also excluded; thus, the possibility of a selection bias cannot be excluded. Under the Japanese insurance system, the base period for which the ICU management fee can be calculated is ≤ 14 days. Patients with severe burns, organ transplantations, and extracorporeal membrane oxygenation were excluded because, as an exception, ICU management fees can be calculated for > 14 days for these patients, and their conditions are highly specific. This may have resulted in a shorter length of ICU stay in both groups. Third, although the effectiveness assessment of ICU management should include mid- to long-term mortality rates and QOL information, ICU mortality rates and in-hospital mortality rates were evaluated as the main clinical outcome in this study because the DPC database only included outcomes at discharge. The ICU and in-hospital mortality rates are important as short-term parameters for evaluating the effectiveness of ICU management. Finally, this study of QALYs was performed with several adjustments based on the methods of earlier studies [17, 18]. The adjustments for life expectancies were performed based on information for patients with acute respiratory failure; therefore, life expectancy was adjusted to be shorter. Although the utility value for QALY ranges from 0.63 to 0.81, it was set to 0.63 in this study, and this may have resulted in an underestimation of the QOL. Such adjustments did not adversely affect the interpretation of the results of cost-effectiveness. However, there is a risk that we may have over-adjusted the results, although a sensitivity analysis was performed to minimize this effect. For a more accurate assessment of QALY, it is necessary to conduct a follow-up evaluation of the mid- to long-term QOL of patients admitted to the ICU. Outpatient visits for patients discharged from the ICU may be a useful follow-up indicator to clarify which staffing models and/or organizational structures improve cost-effectiveness.

## Conclusions

In this observational study covering nationwide acute-care hospitals in Japan, ICU1/2 was associated with lower ICU mortality and in-hospital mortality than ICU3/4. The ICER was < 5 million JPY/QALY; therefore, ICU1/2 is cost-effective. Further investigations are necessary to identify the ICU outcomes and cost-effectiveness with respect to management fees.

### Supplementary Information

**Additional file 1**: Classification of acute care beds in Japan.

**Additional file 2**: Utility values reported for the EQ-5D score for ICU patients.

## Data Availability

The datasets used and/or analyzed during the current study are available from the corresponding author upon reasonable request.

## Abbreviations

CHEERS: Consolidated Health Economic Evaluation Reporting Standards
DPC: Diagnosis procedure combination
EQ-5D: EuroQol 5-dimensions
ICD-10: International Classification of Diagnosis, 10th Revision
ICER: Incremental cost-effectiveness ratio
ICU: Intensive care unit
JPY: Japanese Yen
QALY: Quality-adjusted life-year
QOL: Quality of life
SOFA: Sequential Organ Failure Assessment
