# Supplementary material for: Intensive care unit mortality and cost-effectiveness associated with intensivist staffing: a Japanese nationwide observational study
Source: J Intensive Care. 2023 Dec 4;11:60. doi: 10.1186/s40560-023-00708-w (PMC10694900; doi:10.1186/s40560-023-00708-w)
Supplement: Supplementary file 1 — Additional file 1: Classification of acute care beds in Japan. [file 40560_2023_708_MOESM1_ESM.docx]

**Additional file 1. Classification of acute care beds in Japan**

|  |  | Nurse-to-patient ratio | Human resource criteria | Space criteria |  |
| --- | --- | --- | --- | --- | --- |
| ICUs | ICU management fee 1 | 1:2 | Full-time staff (two or more experienced certified intensivists, certified nurses, and clinical engineers) | At least 20 m^2^ per floor |  |
|  | ICU management fee 2 | 1:2 | Full-time staff (two or more experienced certified intensivists, certified nurses, and clinical engineers) | At least 20 m^2^ per floor, and intensive care for extensive burn patients |  |
|  | ICU management fee 3 | 1:2 | Full-time physician (not necessarily intensivists) | At least 15 m^2^ per floor |  |
|  | ICU management fee 4 | 1:2 | Full-time physician (not necessarily intensivists) | At least 15 m^2^ per floor, and intensive care for extensive burn patients |  |
|  | Emergency and critical care unit management fee 2 | 1:2 | Full-time physician (not necessarily intensivists) | - |  |
|  | Emergency and critical care unit management fee 4 | 1:2 | Full-time physician (not necessarily intensivists) | At least 15 m^2^ per floor, and intensive care for extensive burn patients |  |
|  |  |  |  |  |  |
| HDUs | High care unit management fee 1 | 1:4 | No full-time physician needed | - |  |
|  | High care unit management fee 2 | 1:5 | No full-time physician needed | - |  |
|  | Emergency and critical care unit management fee 1 | 1:4 | Full-time physician (not necessarily intensivists) | - |  |
|  | Emergency and critical care unit management fee 3 | 1:4 | Full-time physician (not necessarily intensivists) | At least 15 m^2^ per floor, and intensive care for extensive burn patients |  |
| General wards |  | 1:7 | No full-time physician needed | - |  |

Neonatal intensive care beds, pediatric intensive care beds, coronary care beds, stroke care beds, and renal care beds.

Note: ICU, intensive care unit; HDU, high-dependency care unit
