# Supplementary material for: Intensive care unit mortality and cost-effectiveness associated with intensivist staffing: a Japanese nationwide observational study
Source: J Intensive Care. 2023 Dec 4;11:60. doi: 10.1186/s40560-023-00708-w (PMC10694900; doi:10.1186/s40560-023-00708-w)
Supplement: Supplementary file 2 — Additional file 2: Utility values reported for the EQ-5D score for ICU patients. [file 40560_2023_708_MOESM2_ESM.docx]

**Additional file 2. Utility values reported for the EQ-5D scores for ICU patients**

| **Authors** | **Population** | **Follow-up** | **EQ-5D score** | **Reference** |
| --- | --- | --- | --- | --- |
| Sznajder et al. | Mixed ICU patients | 6 months | **0.63** | 25 |
|  | Non-surgery |  | **0.61** |  |
|  | Emergency surgery |  | **0.70** |  |
|  | Elective surgery |  | **0.71** |  |
| Granja et al. | Mixed ICU patients | 6 months | 0.81 | 19 |
|  | Non-surgery |  | 0.82 |  |
|  | Emergency surgery |  | 0.77 |  |
|  | Elective surgery |  | 0.76 |  |
| Cuthbertson et Al. | Mixed ICU patients | 3, 6, and 12 months | 0.68 | 21 |
| Åhlström et al. | Mixed ICU patients | Median 2.4 years | 0.68 | 22 |
| Ylipalosaari et Al. | Mixed ICU patients | Median 22 months | 0.72 | 23 |
| Unoki et al. | Mixed ICU patients | 12 months | 0.79 | 24 |
|  | Elective surgery |  | 0.87 |  |
|  | Emergency admission |  | 0.84 |  |

EQ-5D, EuroQol 5-dimensions; ICU, intensive care unit
